# Closing the gap between practice and science in school- and community-based participatory physical literacy promotion: study protocol of the StuPs project

**DOI:** 10.1186/s12889-021-10666-3

**Published:** 2021-04-01

**Authors:** Stefanie Wessely, Dagmar Starke, Simone Weyers, Christine Joisten

**Affiliations:** 1 Department for Physical Activity in Public Health, Institute of Movement and Neurosciences, German Sport University Cologne, Am Sportpark Müngersdorf 6, 50933 Cologne, Germany; 2 Academy of Public Health Services, Kanzerlstr. 4, 40472 Düsseldorf, Germany; 3 Institute of Medical Sociology, Centre for Health and Society, Medical Faculty, Heinrich-Heine-University Düsseldorf, Universitätsstraße 1, 40225 Düsseldorf, Germany

**Keywords:** Physical activity promotion, Physical literacy, Socially deprived urban districts, Community-based approach, Community-based participatory research, Capacity building

## Abstract

**Background:**

The role of physical activity in the promotion of children’s well-being and health is widely known. However, research indicates that the time spent physically exercising and participating in organized sport activities is decreasing among children. Although there is currently no gold standard for promoting sustainable physical activity in children, community-based approaches, particularly those that are multicomponent, appear to be the most successful. The project StuPs: a school- and community-based participatory approach for promoting physical activity in children and their families aims to develop a community-based approach to promoting physical activity by increasing physical literacy among elementary school children and their household members.

**Methods:**

The project is built upon the intervention mapping approach and consists of two periods with an overall duration of 3 years. Period I will last 9 months and include an assessment of needs, wants, strengths, and weaknesses regarding physical activity and health promotion at the community- and school-based level according to the keywords “capacity building” and “physical literacy.” Based on the knowledge gained in this stage, measures for capacity building to promote healthy lifestyles and physical literacy in children will be developed using the community-based participatory research and capacity building approach. In Period II, the measures will be applicated, implemented and evaluated using a pre−/post-design to assess efficacy.

**Discussion:**

Although the efficacy of using community-based and capacity building approaches to reach children is promising, there remains a gap regarding best practices for changing existing structures and habits over the long term and in the sense of promoting physical literacy.

## Background

Increasingly sedentary behavior in children constitutes a growing global challenge, although awareness of the role of physical activity in optimizing children’s health is ubiquitous [1, 2]. Despite the various health benefits of physical activity on physical, psychosocial, cognitive, motor skill, and language development [3], only 20% of children and adolescents worldwide meet the World Health Organization (WHO)‘s physical activity recommendations of 60 min per day [2]. Similar data resulted from a German health survey from 2014 to 2017 conducted by the Robert Koch Institute: only 22.4% of girls and 29.4% of boys met the WHO’s recommendations [4] - a far cry from the German physical activity recommendations of 90 min or more per day among elementary school children [5]. These data have also shown that time spent physically exercising correlates positively with socioeconomic status and negatively with age. Therefore, the challenge remains to develop efficient and sustainable strategies for promoting physical activity among children. The StuPs project: a school- and community-based participatory approach for promoting physical activity in children and their families, was developed for this purpose, particularly in response to “Bewegung und Bewegungsförderung” (“exercise and physical activity promotion”), the German Federal Ministry of Health’s call for funding [6]. The aim of this call was to spread the knowledge of the German recommendations for physical activity in the sense of health in all policies. Currently, there is no gold standard for promoting physical activity in young children, especially among vulnerable groups; despite this, school-based and multicomponent approaches are suspected to be most efficient. Because schools are the optimal environment for reaching a great number of youth demographics, school-based approaches seem to have high efficacy [5]. However, physical activity promotion in schools seems to have a limited impact on children’s movement behavior during their leisure time [7]. Current evidence assumes greater success in preventing childhood obesity if entire communities are involved in creating and promoting healthy environments [8]. The inclusion of additional out-of-school aspects within the scope of multicomponent approaches and the inclusion of families, communities, policies, and stakeholders in a participatory manner is recommended [5, 7]. Participatory approaches, such as community-based participatory research (CBPR) [9], have the potential to generate synergy, reach greater acceptance within the target group, and engender increased engagement [8, 10], especially in combination with capacity building [8, 11]. On a behavioral level, the available evidence supports an approach that promotes physical literacy, a construct that indicates the basis for an active lifestyle and serves as “a primary determinant of health and disease” [12]. Physical literacy thus focuses on skill acquisition. In this way, it is similar to the construct of health literacy, which inculcates the ability to find, understand, evaluate, and apply health information and therefore combines knowledge with motivation and competence [13, 14]. To date, no intervention combining physical literacy with community-based strategies has been conducted. Therefore, the StuPs project aims to close this gap between science and practice by implementing a participatory and interdisciplinary multicomponent approach targeting both schools and communities in two socially deprived areas in Cologne (North Rhine-Westphalia, Germany) within the scope of capacity building for physical literacy promotion.

## Methods

### Study design

**Fig. 1:**
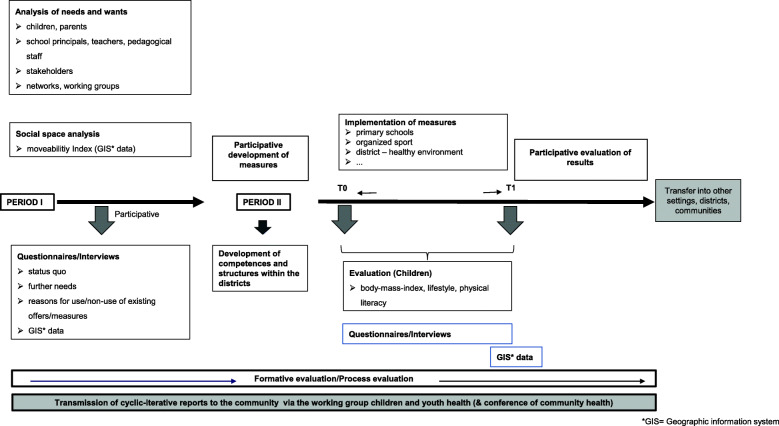
Study Design of the StuPs Project

The StuPs design (Fig. 1) is intended to function as a temporary supporter of the selected districts over the project duration of three years, divided into two periods. The first period (Period I) will be conducted over the course of 9 months. Using a multicomponent, mixed-methods approach, it will identify structures like geographic information system data (GIS), existing capacities, collaborations, and networks to detect needs and wants regarding physical activity promotion in children. At the end of this period, required actions will be deduced. With the knowledge of these required actions, measures to create healthy environments in the sense of capacity building and physical literacy promotion in children will be developed. This procedure will be conducted with the participation of key stakeholders in the two Cologne districts. The intention is to create measures integrable into the routines of the stakeholders, schools, and target group and address individuals as well as their environments. Therefore, these measures will on the one hand address the behaviors of children and their families as well as their districts’ capacities to create healthy environments. On the other hand, they will enable local stakeholders within the topic of health and physical literacy promotion by qualification trainings.

The intention of the second period (Period II) is to implement the developed measures over a 27-month period. Measures will be combined with existing health-related structures in terms of health in all policies. To evaluate efficacy, the status quo will be analyzed in a manner equal to that in Period I to capture changes on structural levels. To investigate behavioral changes, pre- and post-tests of the children’s physical literacy levels will be conducted. Additionally, the German Federal Ministry of Health has employed an external evaluation group [6], to investigate promoters and inhibitors during the StuPs project in the context of the call for funding “Bewegung und Bewegungsförderung”. The aim of the external evaluation is to detect factors of success within community-based approaches. At the end of Period II, a transfer guideline will be developed through an iterative and participatory process to be provided to various districts and communities; this transfer guideline will contain step-by-step instructions, serving as a best practice model, using as few resources as possible. Additionally, it will include information on how to i) capture needs and wants in terms of capacity building and physical literacy promotion in settings, districts, or communities; ii) apply and implement measures; ii) evaluate efficacy; and iv) document procedures to facilitate learning from each other regarding practical experiences. Throughout the entirety of the project, there will be cyclical, iterative reports and dialogues at the community level.

### Participants and recruitment

The approach will be partially randomized. Participating schools from two Cologne districts (Chorweiler and Meschenich) will be recruited for this study. The two districts have been chosen because of their social circumstances, as defined by the Cologne Office for Urban Development and Statistics [15]. The index for social circumstances of urban districts is based on information related to issues such as economic deprivation, political and cultural disadvantage, and health inequality. With the support of the district coordinators responsible for social planning, two elementary schools (one in each district) will be recruited, and local networks will be identified. The elementary schools will provide access to the target group of children, parents, teachers and other pedagogical staff. Any children and parents at the chosen elementary schools will have the opportunity to participate; therefore, the target group will be composed randomly. The identified networks will be used to get in contact with local stakeholders to establish participatory collaborations.

### Intervention mapping

Intervention mapping describes the combination of theory- and evidence-based approaches in a bottom-up procedure containing six steps [16]. Through these six steps, interventions can be designed that are appropriate to the target group and also consider healthy environments [17]. The StuPs project is designed to follow the six stages (Table 1). The target group will be integrated in the identification of needs, wants and development of measures. Additionally, current scientific knowledge and existing behavioral theories will be researched and considered. The six steps of the intervention mapping form the modular structure of the project and emerge in specific work packages.

**Table 1:** Modified Steps of Intervention Mapping within the StuPs Project [16]

Step 1: Assessment of needs, wants and inventory of health-related issues to identify current conditions in terms of capacity building and physical literacy promotion in the selected districts.	
Step 2: Goal specification within the settings/networks with stakeholders in light of the knowledge generated in step 1.	
Step 3: Scientific literature research and comparison with previous results of community-based approaches as well as definitions and instruments to investigate physical literacy, aiming to accompany the theoretical approach with existing knowledge.	
Step 4: Derivation and development of school- and district-related measures based on the knowledge of the previous steps. In this step, the content for the measures conducted later will be created.	
Step 5: Definition of further procedures and implementation of the measures created previously.	
Step 6: Review, evaluation, and development of transfer indicators.	

### Community-based participatory research

The CBPR approach developed by Israel et al. [9] has been decisive in designing the content of the StuPs project. CBPR aims to promote health equity, strategies development, empowerment of a target group, and capacity building within the targeted community [9]. This approach intends to combine scientific, practical, and policy stakeholders as well as different professions. Therefore, the StuPs approach is characterized by a high level of practical orientation to strengthen participatory collaborations between science, practice, and policy. To this end, the CBPR principles by Israel et al. [9] have been modified to promote individual and collective empowerment and the development of expertise and structures in terms of capacity building and physical literacy (Table 2).

**Table 2:** Modified Principles of the Community-Based Participatory Research following Israel et al. [9]

(1) The network sees itself as a unit.	
(2) The network builds on existing strengths and resources.	
(3) The network works transparently and in partnership.	
(4) The network promotes joint development, empowerment, and capacity building.	
(5) The network creates a balance between knowledge generation and interventions for the mutual benefit of all partners.	
(6) The network focuses on specific needs in the context of physical activity promotion, taking into account sociodemographic and gender characteristics as well as systematic geographical data.	
(7) The development of measures and implementation strategies takes place in a cyclical, iterative, and reflexive process.	
(8) All results and developments will be made accessible to all partners; further dissemination and use will also take place in coordination.	
(9) The aim is to create the basis for long-term cooperation and the establishment of the topics as a cross-sectional task of all partners involved (health in all policies), with the intention of sustainably promoting the physical activity (and health) and physical literacy of vulnerable groups.	

### Data management and analysis

Any conducted assessments will be carried out anonymously. The results of Period I will be analyzed and interpreted by conducting qualitative data analysis [18] in the MAXQDA 2020 software. The data resulting from the children and parental pre- and post-tests will be analyzed using the SPSS software version 27.0 (SPSS Inc., Chicago, IL, USA). The significance level α will be set at < .05; confidence intervals will be estimated at 95%. Descriptive analysis will be conducted to identify cross-sectional and long-term changes in physical literacy level, including motor skills, by generating means and variances. Additionally, correlations and influencing factors among variables will be assessed using analysis of covariance and regressions.

## Discussion

Individual habits and behaviors as well as environments (e.g., immediate surroundings, neighborhoods, schools, local policies) have a great impact on a child’s health status [19]. Therefore, healthy environments need to be created, and StuPs has been developed to achieve this aim. This approach is grounded in CBPR [9] and intervention mapping [16], both of which have evidence-based efficacy e.g. in terms of anthropometric and behavioral changes as well as environmental and political outcomes [8, 16, 17]. Previous studies have reported increasing success in terms of intensity of community engagement and participation [8, 10]. In addition, the StuPs approach has been developed following CBPR and intervention mapping to build upon existing knowledge and experiences. Although the rough procedure is comparable with other approaches, StuPs differs in its aim to improve physical literacy in children and their families. Most other community-based approaches have addressed health promotion in general or healthy weight in children specifically [8]. Currently reported physical literacy interventions have mainly been conducted in schools, especially physical education classes [20]. StuPs is unique in its aim to promote physical literacy at the community level, thereby reaching children not only where they learn but also where they live and play. This potential arises from increased scientific attention because physical literacy is assumed to be fundamental in creating physically active lifestyles [20]. According to Carney et al. [12], physical literacy is “a gateway” to lifelong physical activity and is crucial for a positive attitude toward movement. In addition to personal and individual factors such as age, gender, motor skills and psychosocial development, one’s environment is assumed to impact one’s degree of physical literacy [21]. The development of healthy environments - including walkable neighborhoods, opportunities to move and play, and opportunities to participate in sports and physical activity - could motivate and support children and families to develop active and therefore healthy lifestyles. For this reason, the intention of the StuPs project is to spread the knowledge of the physical literacy construct beyond schools and among community stakeholders, which, in combination with capacity building, will create healthy environments and therefore support children in developing more active lifestyles. It is expected that improved physical literacy knowledge among stakeholders will lead to increased readiness to change structures and build capacities for healthy environments for districts’ children. To maintain closure of the gap between science and practice in these efforts, there will be cyclical, iterative reports at the community level within specific working groups. The members of the working groups are practical experts on different community- and district-related issues, and they will support the overall project duration through discussion of any barriers and obstacles that appear. The project will benefit from their expertise and experience as well as incorporate feedback and further impulses and ideas. This will be especially helpful when preparing the transfer guideline to ensure that it is as feasible as possible. With this approach, StuPs intends to contribute knowledge of supporters and barriers to promote sustainable physical activity in the scientific interest of the German Federal Ministry of Health.

## Data Availability

Data sharing is not applicable as this is a study protocol. Study materials such as the developed questionnaires or guidelines are available on reasonable request from the authors.

## Abbreviations

CBPR: Community-Based Participatory Research’
GIS: Geographic Information System
StuPs: ‘Ein schul- und kommunalbasierter Ansatz zur partizipativen Bewegungsförderung von Kindern und deren Familien’
WHO: World Health Organization
